# The Association Between Caregiving Intensity and Caregiver Engagement in Paid Work

**DOI:** 10.1093/geroni/igaf122.2502

**Published:** 2025-12-31

**Authors:** Shan Qu, Qian Song, Jeffrey Burr

**Affiliations:** University of Massachusetts Boston, Boston, Massachusetts, United States; University of Massachusetts Boston, Boston, Massachusetts, United States; University of Massachusetts Boston, Boston, Massachusetts, United States

## Abstract

Framed within the Role Strain Theory, this study examined the relationship between caregiving intensity and engagement in paid work among caregivers aged 25-61 who provided care for older adults. Using data from the National Health and Aging Trends Study and the National Study of Caregiving, we estimated multilevel models to identify within- and between-person effects. Caregiving intensity was measured with number of caregiving hours provided in the previous month (logged for skewness). Paid work was dichotomized as ‘1=not engaged in paid work, 0=engaged in paid work.’ Our results showed that as caregiving hours increased, the likelihood of not engaging in paid work increased for both within-person effects (OR = 1.01, 95%CI=1.00-1.02) and between-person effects (OR = 1.02; 95%CI=1.01-1.04). We also evaluated whether these relationships were non-linear by including a quadric term for hours of caregiving. The results showed a marginally significant non-linear relationship for both within-person effects and between-person effects, demonstrating the possibility that higher levels of caregiving intensity were more impactful in terms of not engaging in paid work than was the case for lower levels of caregiving intensity. Additional results showed that female caregivers and those with higher education were more likely to engage in paid work. Hispanic caregivers were less likely to be engaged in paid than non-Hispanic white caregivers. Sustained high-intensity caregiving increases the likelihood of caregivers not engaging in paid work over time. These findings underscore the need for programs that promote respite care and other services designed to help caregivers remain active in the paid labor market.

